# Governance absorption of volume-based procurement: study of dual-track implementation in a public hospital

**DOI:** 10.3389/fpubh.2026.1834133

**Published:** 2026-06-19

**Authors:** Lei Gao, Rui Mao, Xuan Xiong, Wei Xiong, Xiang An, Lin Wen

**Affiliations:** 1 Department of Medical Equipment, Sichuan Provincial People's Hospital, School of Medicine, University of Electronic Science and Technology of China, Chengdu, China; 2 Department of Pharmacy, Sichuan Provincial People's Hospital, School of Medicine, University of Electronic Science and Technology of China, Chengdu, China; 3 Department of Hepatobiliary and Pancreatic Surgery, Sichuan Provincial People's Hospital, School of Medicine, University of Electronic Science and Technology of China, Chengdu, China; 4 Department of Longquan Campus, Sichuan Provincial People's Hospital, School of Medicine, University of Electronic Science and Technology of China, Chengdu, China

**Keywords:** dual-track implementation, governance absorption, public health, public hospital, volume-based procurement

## Abstract

**Background:**

Volume-based procurement (VBP) is commonly premised on a linear transmission assumption, whereby restructuring procurement rules and price mechanisms is expected to drive clinical substitution. In practice, however, hospital-level change often displays differentiation, phasing, and reversibility, suggesting that policy effects do not simply descend in a linear fashion. This article aims to identify the mechanisms through which policy directives are translated at the hospital organizational level and proposes a governance absorption framework to explain the divergence between execution completion and bedside substitution.

**Methods:**

The study adopts an embedded single-center case study design in a large provincial tertiary public hospital in China. To clarify the boundary of inference, the case was selected for its early and continuous VBP implementation, complete time-stamped governance records, and cross-category variation in consumables. The analysis uses institutional and processual materials, including implementation meeting minutes, cross-departmental coordination records, governance notices, and an execution-tracking ledger covering 37 centralized procurement batches from March 2025 to January 2026. The study aims at analytical rather than statistical generalization.

**Results:**

Procurement directives can be rapidly translated into auditable organizational execution routines, primarily through quota setting, volume-reporting chain construction, and periodic monitoring. By contrast, clinical substitution follows a different accountability logic: outcome responsibility remains borne mainly by frontline clinicians, and product use must remain professionally defensible, particularly in the context of adverse-event review. The study documents three recurrent pathway patterns observed in this case: routine absorption when no major equivalence or workflow exception is recorded; selective adaptation when workflow fit or risk perception remains contested; and temporary suspension (bounded risk retreat) when documented usability or equivalence problems persist across cycles, thereby constraining the substitution process.

**Conclusion:**

The so-called implementation gap is better understood as a governance effect: a structural separation between measurable execution completion and clinical accountability. The governance absorption framework specifies the conditions under which implementation proceeds through routine absorption, selective adaptation, or bounded risk retreat. Achieving sustainable practice change requires completion-oriented goals to be coupled with controversy-closure procedures, shared responsibility arrangements, transition-period support, and dual-track evaluation indicators.

## 1 Introduction

In the context of ongoing global health system reforms, volume-based procurement (VBP) has emerged as a key policy instrument for enhancing the affordability and accessibility of healthcare services (1–3). Its core approach involves reshaping procurement rules, price formation mechanisms, and market entry protocols to drive adjustments in medical product supply chains and utilization behaviors. Although countries such as China, Gulf Cooperation Council, and select European nations differ in their institutional designs and implementation pathways, these reforms commonly rest on an implicit assumption of relatively linear transmission along institutional chains: adjustments in upstream procurement systems are expected to translate into downstream clinical substitution and optimized resource allocation (4, 5).

Existing research has amassed substantial evidence on the macro-level effects of VBP, focusing primarily on price reductions, market restructuring, and aggregate changes in utilization volumes (6, 7). However, compared to these observable outcome metrics, far less detailed explanation exists regarding how such policies are interpreted, internalized, and transformed into routine operational mechanisms within hospital organizations. Mounting empirical observations suggest that policy implementation is not a straightforward top-down progression but entails organizational reinterpretation, reprioritization, and re-embedding (8). Within this dynamic, VBP often manifests a tension-laden pattern: administrative execution exhibits high consistency, whereas clinical adoption tends to be more cautious, limited, and uneven. Consequently, policy effects cannot be viewed merely as direct extrapolations of institutional directives; instead, they must be examined within the internal governance structures of hospitals, where multiple objectives–such as policy compliance, clinical safety, professional accountability, and operational stability–coexist, not inherently aligned but sustained through ongoing coordination and dynamic balancing (9, 10).

Recent evidence from China's National Health City campaign further suggests that public health policy effects are shaped by local administrative capacity, cross-sectoral coordination, and the quality of implementation environments rather than by policy design alone (11). This provides contextual support for examining how hospitals translate and reconfigure policy pressure within their own governance structures.

This issue extends beyond the Chinese context, holding broader international relevance, particularly for public hospital systems under dual pressures of fiscal constraints and intensified regulation (12, 13). On one hand, VBP has become progressively institutionalized in many countries and regions, generating persistent and rigid administrative demands around volume reporting, execution, and auditing. On the other, frontline clinicians continue to bear primary responsibility for treatment outcomes, with product selection required not only to meet cost control and policy mandates but also to align with practical constraints of professional defensibility, workflow continuity, and departmental stability. In this light, assessing policy success solely through price declines or procurement volume shifts often fails to capture underlying mechanisms. To grasp VBP's true policy effects, analysis must delve into hospital interiors, scrutinizing the governance processes that link policy objectives to implementation outcomes (14, 15).

Building on this insight, we do not treat VBP merely as an exogenous policy input but examine it as a governance process unfolding within hospitals (16). To this end, we introduce and apply the analytical lens of “governance absorption” to elucidate how policies are enacted at the organizational level, why they diverge along distinct pathways, and the mechanistic foundations of such divergences. Our central concern is not simply whether policies are effective but how they are produced within organizations, under what conditions implementation pathways bifurcate, and why these bifurcations occur. Centered on this inquiry, we address three research questions:

(1) How are VBP policy directives progressively translated into hospital governance routines?
(2) Under what organizational conditions and contextual factors does this translation manifest as routine absorption, selective adaptation, or temporary suspension (bounded risk retreat)?
(3) What revisions are needed to the conventional understanding of policy transmission as linear from institutional endpoints to clinical practice?

To address these questions, we employ a single-center thick-description case study design, integrating time-stamped institutional texts and execution records to reconstruct how VBP policy signals are translated, monitored, contested, and reconfigured within hospitals. By forging mechanistic explanations between policy goals, organizational execution, and clinical practice, we seek to reveal how hospitals digest policies under multiple constraints and, thereby, propose an operational analytical framework. Our contribution lies not in reaffirming VBP's general policy orientation but in illuminating its specific governance logic at the hospital level, offering comparative explanatory foundations for optimizing VBP policies and improving public hospital governance across diverse institutional settings.

## 2 Analytical framework

VBP is commonly understood as a policy instrument whose basic expectation is that upstream adjustments in institutional rules will induce downstream changes in clinical behavior (17). This expectation embeds a linear transmission logic: policies set procurement rules, hospitals fulfill execution requirements, and clinical practice subsequently shifts toward substitution (18). This logic is useful for identifying macro-level effects such as price reductions, market restructuring, and aggregate changes in utilization. However, it has limited explanatory power for the persistent differentiation, phasing, and reversibility repeatedly observed at implementation sites.

The reason is that hospitals do not function as passive channels of policy transmission. Rather, they are governance organizations that must coordinate multiple objectives. Beyond policy compliance, hospitals must also respond to practical pressures related to clinical safety, professional defensibility, operational continuity, and budgetary constraints (19). In this setting, external procurement signals do not usually move directly into bedside practice. Instead, they are first organizationally translated into internal task decomposition, process monitoring, exception handling, and accountability configurations, and are then sustained through continued follow-up by administrative execution units (12, 20, 21). When the logic of administrative completion comes into tension with frontline clinicians' perceptions of risk, implementation pathways may be adjusted, reconfigured, or even partially rolled back (22).

Building on this understanding, this article introduces the perspective of governance absorption as a refinement of, rather than a replacement for, existing implementation and decoupling perspectives. Governance absorption is defined as the organizational process through which external procurement requirements are converted within hospitals into task decomposition, process monitoring, exception handling, controversy disposition, and accountability configuration. Its theoretical increment lies in specifying the process mechanisms that connect formal compliance and practice-level divergence: translation of policy pressure into auditable routines, screening of substitution through professional defensibility, and bounded organizational adjustment when controversies cannot be closed.

(1) Routine absorption: policy requirements are stably incorporated into standard workflows, producing a relatively durable pattern of execution;
(2) Selective adaptation: policy requirements are broadly implemented, but localized adjustments emerge at the level of specific departments, procedures, or products;
(3) Temporary suspension: when clinical or operational risks are persistently perceived and cannot be resolved in a timely manner, relevant implementation is subject to phased restriction or pause.

Accordingly, the analytical focus shifts from whether policy is broadly effective to how implementation is adjusted in organizational practice, through what specific mechanisms, and under what conditions such adjustment occurs. This shift in perspective helps build testable mechanistic bridges between the assumption of linear policy transmission and hospitals' actual behavior, thereby identifying when policy transmission is sustained, when it weakens, and when it is interrupted.

In this article, governance absorption serves both as an explanatory framework and as an analytical tool. It guides material coding and mechanism identification, while shifting the evaluation of policy implementation from whether outcomes meet formal targets to the tracing of process mechanisms: how compliance is organizationally produced, how clinical boundaries are continually screened, and how controversies are institutionally managed. On this basis, we propose three observable propositions. In institutional environments centered on completion indicators, policy pressure is more likely to be deposited first into auditable execution infrastructures. Where outcome accountability remains primarily anchored at the clinical frontline, bedside substitution will continue to be constrained and filtered by thresholds of professional defensibility. When controversies over equivalence, usability, or workflow fit persist across cycles without sufficient escalation and closure, organizations are more likely to resort to bounded risk retreat in order to maintain overall continuity while containing localized risks.

To formalize the framework more explicitly, the three pathways are treated as mechanism-based propositions rather than as merely descriptive labels. Proposition 1: when execution requirements can be incorporated into auditable routines and professional defensibility is not substantially threatened, implementation tends toward routine absorption. Proposition 2: when auditable execution can continue but workflow fit, product equivalence, or risk perception remains locally contested, implementation tends toward selective adaptation. Proposition 3: when controversies persist across cycles and responsibility for adverse outcomes remains concentrated at the clinical frontline, implementation tends toward bounded risk retreat through temporary suspension, exclusion, or conditional re-inclusion.

## 3 Methods

### 3.1 Study design and case selection

This study adopts a single-center case study design with thick description and incorporates embedded comparison across two dimensions: product categories and governance interfaces (23, 24). The design is intended to address how VBP is translated into organizational practice within hospitals, and under what conditions implementation diverges into routine absorption, selective adaptation, and temporary suspension (bounded risk retreat).

The case hospital was selected on the basis of three criteria. First, it entered the VBP implementation sequence relatively early and maintained continuous execution records. Second, it retained contemporaneous institutional records sufficient to support process tracing. Third, implementation trajectories differed clearly across product categories, making comparative analysis possible.

The case hospital is informative for mechanism-building purposes because it combines high regulatory exposure, large-scale public hospital functions, and heterogeneous clinical use scenarios for procured consumables. At the same time, it is not treated as statistically representative of all Chinese hospitals. The study therefore uses the case to examine mechanisms that may be analytically transferable under specified institutional conditions: quantifiable completion pressure, traceable procurement governance, and continuing clinical accountability for substitution outcomes.

The analytical time window covers the hospital's most recent completed closed-loop cycle most directly related to VBP policy, namely volume reporting, execution, review/rectification, and continuation/adjustment, in order to preserve both evidentiary recency and process completeness. Specifically, the study period runs from March 2025 to January 2026, corresponding to batches 1 through 37 recorded in the hospital's internal VBP implementation ledger. All materials used in the analysis are formal institutional records generated and archived during the study period. Each batch-level chain of volume reporting, execution, review/rectification, and continuation/adjustment is treated as the minimum unit of process tracing.

### 3.2 Data sources and inclusion criteria

Empirical materials were drawn primarily from the hospital's internal administrative and operational records, including volume-reporting templates, execution ledgers, implementation meeting minutes, departmental notices, and routine management briefings. Where available, these materials were supplemented by category-specific volume-reporting documents and accompanying explanatory materials. Because the study focuses on organizational implementation mechanisms, priority was given to time-stamped institutional records in order to support process tracing, and cross-checking across document types and organizational levels was used to reduce recall bias.

The empirical corpus included four types of materials: implementation meeting minutes and coordination records, hospital governance notices and operational rules, supplier rectification and problem-feedback materials, and an execution-tracking ledger covering 37 centralized procurement batches. These materials were used not as isolated illustrations but as a linked process chain. The meeting and coordination records identified decision points and controversy-handling procedures; governance notices identified formal rules and responsibilities; rectification materials identified unresolved usability or equivalence disputes; and the execution ledger identified batch-level continuity, reporting progress, and follow-up status across time.

Documents were included if they provided directly verifiable evidence for at least one of the following governance domains: goal setting, execution monitoring, exception handling, or accountability configurations. Documents were excluded if they lacked a clear date or version identifier, duplicated existing content, or had no direct relevance to implementation decisions. In total, 11 items were included: 2 meeting minutes, 1 coordination record, 1 governance notice or bulletin, 4 execution ledgers or tracking tables, and 3 supplementary materials. All included materials carried explicit date or version information.

To enhance the transparency and traceability of the evidence chain, Table 1 maps each material type to the governance processes it records, the findings it supports, and the corresponding anchor points in the supplementary appendices. Section-specific evidence anchors are provided in Table 1 and Appendices A-B.

**Table 1:** Evidence–to–claim mapping: empirical materials, governance processes, and analytical contribution.

Empirical material type (examples)	Source/format	Governance process documented	Analytical contribution (claim)	Linked Findings section	Supplementary anchor
Minutes of provincial VBP implementation discussions (2025)	Internal hospital meeting minutes	Cross–department negotiation of reporting volumes across product categories; differentiated handling and feasibility concerns	Policy requirements are organizationally translated into internal implementation arrangements rather than uniformly enacted at point of care	4.2	B1
Hospital weekly meeting slides on centrally procured ultrasonic devices	Internal PPT (medical equipment department)	Escalation of low completion progress; hierarchical instruction; linkage to departmental performance	Compliance pressure is produced through managerial instruments and auditable targets	4.2	B1
Procurement and governance notices (eg, quota allocation based on historical use; assigned monitoring responsibilities)	Administrative notices/circulars	Formalization of quotas, reporting cadence, and monitoring chains	Administrative infrastructure converts external policy into routinized internal completion workflow	4.2	B1/B4
Item–level execution ledgers across procurement batches (2025–2026 tracker, 37 batches)	Structured tracking sheets/ledgers	Batch–level progress tracking by product, contract period, and execution status	Completion is rendered legible and auditable at scale, without implying transfer of clinical accountability	4.1	B5
Internal suitability/adaptation records for consumables (e.g., sutures, vacuum blood collection tubes)	Structured internal governance records	Compatibility appraisal, staged trial use, differentiated adoption by risk profile	Selective adaptation emerges when implementation is conditioned by workflow fit and perceived risk	4.3	B4, B3
Coordination records on disposable oxygen tubing	Internal coordination notes/minutes	Repeated frontline usability feedback; multiple rectification rounds; bounded implementation decisions	Accountability for risk constrains substitution and contributes to conditional continuation or bounded retreat	4.1, 4.3	B2, B6
Cross–unit coordination records (procurement–medical affairs–clinical–nursing)	Internal records across units	Inter–unit alignment on completion, exception handling, and responsibility allocation	Absorption outcomes are co–produced across governance interfaces, not by a single department	4.2, 4.3	B4, B5
Systematized experiential observations (de–identified analytic memos)	Analytic memos (non–evidentiary)	Pattern spotting and document tracing support	Used only to guide retrieval/comparison and analytic checking; not standalone empirical evidence for findings claims	Methods only	N/A

### 3.3 Analytical strategy and pathway identification

Data analysis followed a two-stage strategy that combined concept-driven coding with mechanism-oriented identification. In the first stage, initial coding was guided by the pre-specified analytical framework and organized around organizational translation, monitoring chains, exception rules, and accountability configurations. In the second stage, coding shifted to an inductive mode in order to identify boundary conditions and process variation beyond the initial framework. On this basis, cross-category matrix comparison was used to synthesize the condition combinations and triggering mechanisms associated with the three response modes: routine absorption, selective adaptation, and temporary suspension.

To improve reproducibility, coding decisions were documented in a category-by-category matrix. For each product category, the authors recorded the relevant policy requirement, departmental responsibility arrangement, evidence of routine monitoring, exception clauses or scope restrictions, controversy records, supplier rectification status, and final implementation pathway. Disagreements were resolved by returning to the original time-stamped records and by checking whether the pathway assignment was supported by at least two types of documents. This procedure was intended to reduce reliance on single-document interpretation and to make the derivation of mechanisms more transparent.

The explanatory analysis centered on two critical nodes. The first was the conversion of external procurement requirements into internal governance tasks, including task decomposition, the setting of volume-reporting cadence, the assignment of monitoring responsibilities, and departmental accountability configurations. The second concerned why bedside substitution, under conditions in which outcome accountability remained anchored to the clinical frontline, displayed phasing, caution, and reversibility. Mechanism identification focused on three main threads: organizational translation, clinical screening, and controversy reconfiguration through bounded risk retreat. Cross-category matrix comparison was then used to examine the condition combinations underlying the three forms of implementation.

Classification of the three implementation forms followed consistent rules. A product category was coded as routine absorption when, across consecutive cycles, it showed templated volume-reporting arrangements, routinized monitoring mechanisms, and no significant exception clauses in bedside substitution. It was coded as selective adaptation when implementation continued largely through the standard template but incorporated exception clauses, scope restrictions, scenario limitations, or tiered authorization at the level of departments, procedures, or products. It was coded as temporary suspension (bounded risk retreat) when controversies persisted across cycles and were explicitly written into conditional provisions such as “temporary suspension,” “exclusion,” or “re-inclusion after rectification targets are met.”

To make cross-category comparison explicit, each pathway was checked against three observable dimensions: whether execution targets were decomposed into routine monitoring objects, whether bedside substitution was accepted without major exception clauses, and whether unresolved controversy led to restriction, conditional continuation, or temporary suspension. This coding strategy reduces the risk of over-generalizing from a single product case.

For transparency, pathway assignment was linked to document-level evidence rather than inferred from outcome labels alone. For example, repeated oxygen tubing leakage feedback and three rounds of unresolved supplier rectification were first coded as sustained usability controversy and unresolved rectification, and then classified as bounded risk retreat. Historical-use-based quota allocation and joint monitoring for sutures were coded as task decomposition and routine monitoring, supporting routine absorption. Model mapping for vacuum blood collection tubes was coded as specification adjustment within a continuing reporting framework, supporting selective adaptation.

### 3.4 Researcher positionality and reflexive control

Some authors had previously been directly involved in procurement coordination work at the study hospital. This positionality facilitated access to contemporaneous, continuous, and granular internal organizational materials, but it also carried the risk of introducing insider interpretive bias. To reduce this risk, the study adopted three measures: first, dated institutional documents were used as the primary evidence, with triangulation across document types and organizational levels; second, for materials produced by the authors' own department, initial coding was conducted independently by team members who had not been directly involved, followed by joint deliberation; third, the entire analytical process preserved a time-stamped audit trail of coding decisions, version revisions, and the resolution of disagreements.

We also strengthened bias control in three ways. First, insider knowledge was used mainly to reconstruct institutional sequences and was checked against written records rather than treated as independent evidence. Second, coding was cross-checked by authors from different functional backgrounds, including equipment management, pharmacy, clinical departments, and hospital administration. Third, claims about patient outcomes, product superiority, or long-term cost effects were deliberately excluded from the scope of inference because the available materials were designed to trace governance processes rather than clinical endpoints.

In addition, de-identified experience-based memoranda were used only for document tracking and pattern verification and were not treated as independent empirical evidence.

### 3.5 Ethics and data governance

All documents were de-identified before analysis. No individual patient data were collected. The object of analysis was restricted to organizational implementation mechanisms, and no causal inference was made regarding clinical efficacy or patient outcomes. Conclusions at the operational level are reported only within the evidentiary bounds supported by contemporaneous institutional documents. The acquisition, storage, and use of materials all followed institutional authorization and internal approval procedures.

## 4 Findings

To facilitate presentation, we juxtapose the linear transmission expectations implicit in policy documents with the execution arrangements, monitoring cadence, exception clauses, and controversy handling recorded in hospital documents (evidence sources and anchors are provided in Table 2). Three recurring empirical patterns can be observed across the materials. First, volume reporting and execution have become highly institutionalized and continuously documented. Second, when controversies arise over equivalence, usability, or workflow fit, bedside use tends to become more conservative and conditional. Third, when such controversies persist across cycles, hospital-level decisions more often adopt a segmented advancement strategy that preserves the baseline of auditable completion, narrows the scope of contested substitution, and ties further advancement to evidence of rectification.

**Table 2:** From assumed policy transmission to observed governance processes.

Findings section	Policy design assumption	Expected transmission pathway	What we observed in hospital governance	Mechanism–based interpretation
4.1 What remains anchored in clinical work	Procurement implementation will shift routine clinical choice through mandated use	Lower prices and access rules lead to direct bedside substitution	Reporting and execution became more visible and auditable, but responsibility for outcomes remained with frontline clinicians; use decisions continued to be filtered through professional defensibility	Accountability remains clinically anchored, so completion pressure does not automatically produce unconditional substitution
4.2 Organizational translation into completion infrastructure	Economic incentives and procurement mandates will be internalized by clinicians through routine practice change	Cost and access signals are absorbed in clinical routines with limited mediation	Mandates were translated into internal infrastructure: quota assignment, standardized reporting ratios, tracker updates, cross–department monitoring, and performance–linked follow–up	Policy is first absorbed organizationally, through administrative routines that prioritize checkable completion
4.3 Risk–bounded retreat under contested substitution	Standardization and interchangeability will produce uniform uptake across departments	Similar products are used similarly once included in policy pathways	Under persistent usability/equivalence concerns, implementation was narrowed or staged: contested items were bounded, existing pathways preserved, and continuation made conditional on corrective evidence	Implementation is conditional under uncertainty; hospitals manage risk by sequencing and bounding substitution rather than reversing policy

The revised presentation distinguishes two layers of evidence. First, the execution layer records whether policy tasks were decomposed, monitored, and reported. Second, the clinical transition layer records whether product equivalence, workflow fit, and risk responsibility were sufficiently settled for bedside substitution. The comparison below shows how the same procurement pressure produced different pathways across product categories.

Table 3 summarizes the evidence logic used to distinguish routine absorption, selective adaptation, and bounded risk retreat; it specifies the execution evidence, clinical-governance signal, documentary support, and interpretive boundary for each pathway.

**Table 3:** Evidence logic for distinguishing the three implementation pathways.

Evidence type	Routine absorption	Selective adaptation	Bounded risk retreat
Execution infrastructure	Historical–use–based target setting, departmental decomposition, routine monitoring.	Standard monitoring continued, but specifications, models, or use scenarios were locally adjusted.	Reporting and rectification were retained, but the contested item was temporarily excluded or conditionally advanced.
Clinical–governance signal	No major exception clause or unresolved workflow controversy was recorded in the included materials.	Use remained clinically possible, but professional defensibility required narrowed scope or conditional use.	Repeated usability complaints or unresolved equivalence concerns prevented controversy closure.
Documentary support	Implementation minutes, volume–reporting targets, and joint monitoring records.	Specification–adjustment records, departmental feedback, and conditional implementation notes.	Problem feedback, repeated supplier rectification, departmental reports, and exclusion or re–inclusion clauses.
Interpretive boundary	Suggests possible coupling between administrative completion and clinical substitution where no major clinical–governance controversy is recorded.	Suggests organized adaptation rather than non–compliance.	Suggests reversible and object–bounded risk containment rather than wholesale rejection of VBP.

### 4.1 Enhanced execution visibility and the retention of clinical accountability

Evidence across materials shows that VBP made more visible and traceable the hospital's volume-reporting, submission, and monitoring systems, making execution completion continuously verifiable and traceable across batches. The 2025–2026 execution ledger covers 37 batches and records contract periods and execution status on a continuous basis, indicating that auditable completion at the administrative level had become a recurrent administrative monitoring arrangement during the study period.

Auditable completion, however, does not imply a transfer of clinical accountability. Coordination records and review materials suggest that when controversies over usability or equivalence persist, clinical teams revert to standards of professional defensibility, particularly in the context of reviewing potential adverse events. The oxygen tubing case illustrates this clearly. After three rounds of supplier rectification, more than 20 departments continued to report water leakage. The item was therefore excluded from the current round of volume reporting, and any subsequent advancement was explicitly tied to rectification results at the end of the contract period (see Table 1).

Table 4 applies this evidence logic to the main product categories, showing which documents support the classification of sutures, syringe or vacuum blood collection tube adjustments, and oxygen tubing into different implementation pathways.

**Table 4:** Cross–category evidence matrix linking product cases to implementation pathways.

Product category	Pathway	Process evidence	Clinical–governance condition	Mechanism illustrated
Sutures	Routine absorption	Volume–reporting target set at 80% of historical use; tasks decomposed to departments; Medical Equipment Department and Medical Affairs Department jointly monitored execution.	Equivalence and workflow fit were sufficiently accepted; implementation entered standard monitoring without major exception clauses.	Suggests how administrative decomposition may support relatively stable use when no major defensibility controversy is documented.
Syringe specification/vacuum blood collection tube adjustments	Selective adaptation	Standard reporting and monitoring continued, but specific specifications or models were adjusted according to departmental use scenarios.	Workflow fit generated localized exceptions rather than full suspension.	Suggests that implementation heterogeneity may reflect organized adaptation rather than simple implementation failure.
Oxygen tubing	Bounded risk retreat	After three rounds of supplier rectification, more than 20 departments still reported water leakage; the item was excluded from the current round and re–inclusion was tied to later rectification results.	Usability controversy remained unresolved and outcome responsibility stayed concentrated at the clinical frontline.	Suggests how this organization preserved overall policy continuity while containing a documented localized usability risk.

This suggests that under conditions of asymmetric accountability structures, the organization can continue to advance execution completion, while bedside substitution remains conditional and cautious.

### 4.2 Policy translation and the institutionalization of execution infrastructure

The included documents do not support an inference that VBP directly and uniformly drove bedside substitution. Instead, it is first translated into execution objects that can be assigned, tracked, and reviewed. In practice, this takes the form of task-volume setting, standardized volume-reporting ratios, periodic inspections, and follow-up linked to performance management.

A 2025 implementation minute suggests that in the sutures category, volume-reporting targets were set at 80% of historical usage and then decomposed to departments, with monitoring jointly undertaken by the Medical Equipment Department and the Medical Affairs Department (see Table 1). This coordination mechanism was subsequently extended to other consumables and came to cover both nursing and non-nursing pathways. Materials from weekly meetings on ultrasonic scalpels further suggest that when contracts were approaching expiration and progress lagged behind, management initiated escalation measures and incorporated execution completion into departmental performance-linked management.

Taken together, these findings suggest that the immediate effect of procurement policy at the hospital level lies first in the institutionalized production of execution completion, rather than in the automatic alignment of bedside practice.

### 4.3 The mechanism of bounded risk retreat under conditions of controversy

When product equivalence or usability remains contested over time, the implementation process does not break down as a whole or reverse policy direction. Instead, it undergoes an organized reconfiguration. Cross-category evidence suggests a recurrent combination of strategies: maintaining the baseline of auditable completion, narrowing the scope of contested substitution, and tying further advancement to evidence of rectification (see Table 1).

The oxygen tubing case offers a relatively complete illustration of this mechanism. Against the background of persistent feedback on water leakage, the organization adopted parallel strategies of continuity and containment. In the current round of volume reporting, existing brands were retained, task advancement remained anchored to the historical template, and monitoring responsibilities remained unchanged, while a precondition was set that further advancement would depend on rectification meeting the required standard. Similar logics also appeared in categories such as syringe specification adjustment and vacuum blood collection tube model adjustment.

In process terms, bounded risk retreat followed a sequence of problem feedback, supplier rectification, repeated departmental reporting, and conditional exclusion. The key point is that temporary suspension was not a discretionary refusal of VBP. It was a recorded governance response triggered when evidence of usability problems could not be closed within the existing escalation cycle.

Bounded risk retreat, therefore, does not amount to policy rejection. Rather, it is an organizational response to uncertainty that preserves implementation stability and organizational controllability through segmented, layered, and conditional strategies. On this basis, the article further discusses the implications of this mechanism for revising explanations of implementation gaps and for informing future policy design.

## 5 Discussion

### 5.1 From implementation gap to dual-track implementation: a governance explanation of uneven substitution

The first issue this study seeks to clarify is the explanatory boundary of the commonly invoked judgment of an implementation gap in hospital settings. If the sole criterion is whether policy objectives and clinical substitution move in synchrony, then many phenomena documented in this case could readily be classified as under-execution. However, once policy implementation is placed back into the hospital's actual governance structure, that characterization becomes analytically insufficient (25, 26).

After entering the hospital, volume-based procurement was not observed in the documents as acting directly and uniformly at the bedside. It is first organizationally translated into an execution infrastructure that is decomposable, monitorable, assessable, and accountable. Volume-reporting templates, progress review routines, cross-departmental coordination chains, contract-period management, and performance-linked arrangements are not ancillary to implementation; they are central components of the implementation process documented in this case (1, 27, 28). In this sense, the policy is first absorbed into an execution infrastructure capable of stably producing visible completion.

The problem is that visible completion and clinically usable substitution do not share the same accountability logic. The administrative system can stably complete procurement tasks without requiring the clinical system to substitute synchronously. Frontline clinicians operate under a different set of constraints, including outcome responsibility, risk traceability, event review, and professional scrutiny. These constraints do not take task completion as their endpoint; they are bounded instead by whether professional defensibility can be sustained. When equivalence, usability, or workflow fit remains uncertain, clinical teams tend to maintain cautious and conditional use rather than conform to expectations of linear substitution (19).

This gives rise to two parallel yet coupled governance tracks within the hospital: an administrative track that continuously advances auditable execution completion, and a clinical track that continuously screens for professional defensibility. These two tracks do not cancel each other out. Rather, they perform different governance tasks and generate outcomes on different temporal scales. The former emphasizes outputs that are visible within a reporting cycle, whereas the latter emphasizes responsibilities that remain defensible across clinical scenarios.

This dual-track arrangement may be understood as an organizational response to uneven accountability. Completion indicators move through managerial chains, whereas responsibility for usability, adverse-event interpretation, and clinical defensibility remains closer to the clinical frontline. When closure points and restoration conditions are unclear, auditable completion can continue while clinicians maintain defensibility boundaries. Bounded risk retreat is therefore interpreted here as a limited compromise between these two logics.

Dual-track implementation should therefore not be read simply as policy hollowing out. In this case, it suggests an organizational accommodation between externally verifiable requirements and internal risk boundaries. This interpretation helps explain why implementation should not be reduced to a single completion indicator or to implementation failure alone.

### 5.2 Cross-category pathway types: heterogeneity as conditioned differentiation rather than noise

Extending the logic of dual-track implementation and its underlying allocation of responsibility, once the analytical lens moves from a single product category to multiple categories, what comes into view is not a collection of isolated cases but a recurrent pattern of conditioned differentiation. Divergent implementation trajectories across product categories do not arise because some departments are more supportive of the policy or some teams place greater emphasis on cautious substitution. Rather, each category, once it enters implementation, must confront two thresholds simultaneously. The first is a completion-logic threshold, which requires the candidate product to be incorporated into the organizational chain of volume reporting, monitoring, review, and accountability (29). The second is a professional-defensibility threshold, which requires product choice in real clinical settings to rest on a risk-control basis that is interpretable, acceptable, and traceable (30).

When the first threshold is compatible with the second, the pathway tends toward routine absorption. Under such conditions, administrative advancement does not significantly increase the clinical cost of defensibility, and clinical adoption does not weaken execution stability. The two tracks reinforce each other in the same direction, and policy transmission therefore displays a relatively high degree of consistency.

When the first threshold remains stable but the second comes under pressure, the pathway shifts toward selective adaptation. The organizational system continues to maintain auditable outputs of execution completion, while the clinical side controls risk exposure through scope contraction, scenario restriction, conditional triggering, and related measures. The result is a parallel state in which on-paper advancement continues while bedside change remains constrained.

When the second threshold remains under sustained high pressure and controversy cannot be closed in the short term through escalation mechanisms, the pathway moves into temporary suspension. Its core mechanism is bounded risk retreat: not a system-wide interruption, but a reversible adjustment applied to contested elements while overall execution continuity is preserved.

This implies that cross-category heterogeneity is, in essence, the outward expression of differences in combinations of constraints rather than implementation noise. Precisely for this reason, judging the depth of policy transformation primarily through aggregate indicators involves an inherent blind spot. Aggregate indicators can accurately record the output of the completion track, but they are less capable of capturing the frictions, waiting periods, and oscillations characteristic of the clinical screening track. Unless both tracks are incorporated into evaluation simultaneously, policy performance may appear strong statistically while change in practice remains uneven, ultimately producing a persistent divergence between formal assessment and frontline perception.

### 5.3 Mechanistic contribution and theoretical advancement: from outcome decoupling to processual governance absorption

The theoretical contribution of this article lies not in reaffirming the familiar finding that formal compliance and substantive practice may diverge, but in advancing the analytical focus toward how such divergence is continuously produced through organizational processes. In other words, the article does not stop at outcome classification; it specifies a meso-level mechanistic chain.

Once policy requirements are organizationally translated into the execution infrastructure, the organization can stably produce auditable states of completion. At the same time, the clinical side uses professional defensibility as a filter that reshapes the boundaries of substitution. When controversy persists across cycles, the organization relies on bounded risk retreat to preserve system continuity while containing localized risk. This mechanistic chain explains why strong execution and weak convergence can emerge simultaneously, coexist over time, and do so without internal contradiction.

Compared with classical decoupling theory, the concept of governance absorption is used here as a meso-level process lens. Decoupling theory is well suited to identifying states: organizations may appear coherent while practice diverges (31, 32). Governance absorption adds a mechanism-oriented account: how coherence is produced, when divergence is triggered, and how divergence is organizationally managed without generating system instability. This is especially important in hospitals, which are characterized by high accountability, strong professionalism, and tightly structured workflows. In such organizations, compliance is not a single act but an operational system jointly sustained by templates, timing points, responsibility arrangements, evidentiary requirements, and escalation pathways. To treat this system as merely a layer of formal decoration is to underestimate its governance function; to equate it directly with clinical substitution is to overestimate its capacity to penetrate practice. Governance absorption is useful for examining the analytical space between these two risks of interpretation. It recognizes the real productive capacity of the execution system while also revealing the institutional friction surface between that system and bedside practice.

This distinction clarifies the theoretical increment of the study. Decoupling theory helps identify the coexistence of formal conformity and practice-level divergence, whereas governance absorption explains the organizational work through which this coexistence is produced, stabilized, and sometimes reversed. It therefore adds a processual account of translation, screening, controversy closure, and risk redistribution inside hospital governance.

More cautiously, this mechanism may be analytically transferable to settings that share similar institutional conditions. When a policy implementation setting simultaneously exhibits three observable signs - institutionalized execution infrastructure, continued screening through professional defensibility, and bounded risk retreat under unresolved controversy - these features may be consistent with governance absorption. However, this inference requires further comparative testing. The framework is therefore offered as a mechanism-oriented sensitizing concept rather than a general explanatory theory derived from a single case.

### 5.4 Boundary conditions and policy counterfactuals

The mechanisms identified here do not operate with equal force across all settings. Their explanatory power depends on two coexisting boundary conditions. The first is a high degree of reliance by regulatory and assessment systems on quantifiable completion. The second is the continued primary bearing of outcome responsibility by frontline clinicians (33, 34). The former explains why organizations prioritize investment in execution infrastructure; the latter explains why clinicians continue to maintain defensibility screening. When these two conditions are layered together, dual-track implementation is at its most stable. When either condition relaxes, both the intensity and the direction of pathway differentiation may change.

If quantitative regulatory pressure weakens, organizational investment in completion logic declines, and implementation becomes more likely to rely on local negotiation and informal adaptation. If outcome responsibility is more fully institutionalized as a shared responsibility, the clinical cost of defensibility falls, the probability of routine absorption rises, and the likelihood of selective adaptation and temporary suspension declines.

This boundary judgment suggests a practical implication for policy optimization: compliance should include transition quality as well as volume reporting, contract enforcement, and progress monitoring. Trial-use protocols, controversy-escalation pathways, review milestones, restoration conditions, and completion indicators could all be made recordable and reviewable.

The purpose is not to add procedural burden, but to make transition governance less dependent on *ad hoc* meetings and case-by-case coordination.

Such a shift may better connect completion logic with professional defensibility before controversy accumulates. Clear interfaces and escalation pathways can help ensure that execution completion and transition quality are reviewed together rather than in separate cycles.

Accordingly, the next stage of VBP governance should consider how contentious clinical transitions can be made transparent and accountable while preserving completion discipline.

The mechanism may also be relevant beyond this specific setting, but only under comparable institutional conditions. Procurement or payment reforms may display similar dual-track rhythms when externally verifiable completion pressure coexists with continued professional responsibility at the bedside. This comparison remains a hypothesis for further empirical testing.

The policy implication is therefore not simply to intensify execution discipline, but to include transition governance within the definition of compliance so that completion and transition quality can be recorded and reviewed together.

## 6 Limitations and transferability

This study is a single-center case study with thick description. It focuses on the mechanisms of organizational translation and governance absorption through which volume-based procurement is implemented within hospitals. It does not attempt causal identification with respect to patient outcomes, product-level clinical superiority or inferiority, or long-term cost effects. Its conclusions are therefore not intended for statistical generalization beyond the case.

Because the empirical materials were institutional and processual, the study cannot assess whether the observed pathways improved or worsened patient outcomes, clinical quality, or product-level safety. The practical implications therefore concern the design of transition governance, controversy closure, and responsibility sharing, rather than claims about clinical effectiveness.

Accordingly, the conclusions should not be read as claims about the average effect of VBP across hospitals or product markets. Their applicable boundary is narrower: the framework is most useful where external assessment relies heavily on quantifiable completion indicators, while bedside outcome responsibility remains primarily borne by professional groups.

The governance maturity, organizational history, and management style of the case hospital may shape the specific tempo and forms through which the observed processes unfold. The evidence is drawn primarily from formal institutional materials with time stamps. This provides advantages in terms of process traceability and lower recall bias, but captures undocumented informal negotiation and micro-level adjustment only to a limited extent.

Within these boundaries, the article offers a limited but explicit form of analytic transferability. The premise for extrapolation lies not in similarity of hospital type, but in isomorphism in institutional constraints—specifically, the coexistence of quantifiable requirements for execution completion and the continued primary bearing of outcome responsibility by the clinical frontline. Under these conditions, policy implementation is more likely to display three features: execution completion is readily institutionalized, bedside substitution remains subject to ongoing screening through professional defensibility, and selective adaptation or temporary suspension (bounded risk retreat) may emerge under conditions of controversy.

## 7 Policy and practice implications

The findings of this study suggest that, if volume-based procurement is to move from target attainment to practice convergence, the key does not lie in further intensifying quota pressure. Rather, it lies in institutionalizing transition governance as part of compliance. Four practical directions follow from this argument:

Table 5 translates these implications into operational components by linking each policy mechanism to responsible actors, time nodes, indicators, and expected governance effects.

**Table 5:** Operationalizing transition governance in VBP implementation.

Policy mechanism	Responsible actors	Time node	Operational indicators	Expected governance effect
Transition governance in compliance	Hospital procurement office, medical affairs department, clinical departments, suppliers	Before reporting, during trial use, and before contract renewal	Trial–use protocol completion rate; feedback form return rate; restoration criteria documented	Substitution proceeds with recorded evidence rather than informal tolerance of uncertainty.
Controversy escalation and closure	Hospital VBP coordination group with clinical expert input	Within a defined cycle after repeated complaints or adverse–event review signals	Time to controversy grading; closure rate; number of unresolved issues carried into the next cycle	Controversies are converted into accountable decisions rather than prolonged ambiguity.
Risk and responsibility sharing	Hospital leadership, clinical departments, procurement office, suppliers	During transition periods for contested categories	Supplier rectification response time; joint review records; temporary exception approval rate	Clinical defensibility pressure is reduced without weakening execution discipline.
Dual–track evaluation	Regulators and hospital performance management units	Quarterly or contract–period review	Completion rate plus transition–quality indicators such as usability attainment and complaint closure	Policy performance reflects both auditable completion and bedside convergence.

(1) Incorporate transition governance into the definition of compliance.

Write trial-use protocols, feedback specifications, review milestones, restoration conditions, and evidentiary standards into recordable and verifiable workflows, so that bedside substitution is supported by executable transition pathways.

(2) Build an escalation architecture for controversy closure.

Clarify controversy grading, responsible actors, disposition timelines, evidentiary requirements, and decision authority, so that controversies are not left suspended across cycles and repeatedly drain clinical defensibility.

(3) Promote institutional co-sharing of risk and responsibility.

Without weakening completion discipline, provide transition-period support and risk-sharing arrangements for contested categories, thereby reducing the structural pressure through which risk is left by default at the clinical end.

(4) Dual-track evaluation indicators.

In addition to completion rates and progress rates, introduce transition-quality indicators in parallel, such as timeliness of problem closure, conversion of trial-use feedback, usability attainment, and closure rates for adverse-event or complaint review, in order to reduce the systematic gap between on-paper completion and bedside convergence.

These indicators are intended to improve organizational transition governance. They should not be interpreted as substitutes for clinical evaluation, regulatory assessment, or product-level safety and effectiveness evidence.

## 8 Conclusion

The empirical materials in this study suggest that, once volume-based procurement enters the hospital, what happens first is not bedside substitution but governance reordering. External policy requirements are progressively rewritten into an internally operable task system and are continually sedimented into auditable execution routines through templates, cadence, monitoring, and accountability mechanisms. Change on the clinical side takes the form of frontline teams' continued scrutiny of substitution options through the lens of professional defensibility under conditions of outcome responsibility. These two rhythms were documented as moving forward in parallel and producing differentiated trajectories in the included materials. In this case, some categories were classified as routine absorption, some as selective adaptation, and some as temporary suspension (bounded risk retreat) when controversies remain unresolved.

By shifting the implementation gap from a judgment about capacity to an explanation of mechanism, this article suggests that the observed pattern reflects the reality of dual-track governance under coexisting accountability structures more than any simple lack of willingness or intensity in policy execution.

On the basis of this mechanistic account, policy optimization should not be limited to tightening quota and progress requirements. It should instead incorporate transition governance into the compliance structure, so that execution completion and transition quality become simultaneously recordable, verifiable, and accountable within a unified accountability architecture.

## Data Availability

The original contributions presented in the study are included in the article/supplementary material, further inquiries can be directed to the corresponding author.
